# Clinical Development of Regenerative Medicine Targeted for Intervertebral Disc Disease

**DOI:** 10.3390/medicina58020267

**Published:** 2022-02-10

**Authors:** Daisuke Sakai, Jordy Schol, Masahiko Watanabe

**Affiliations:** Department of Orthopaedic Surgery, Surgical Science, School of Medicine, Tokai University, Isehara 259-1193, Japan; schol.jordy@gmail.com (J.S.); masahiko@is.icc.u-tokai.ac.jp (M.W.)

**Keywords:** cell therapy, stem cells, intervertebral disc, degeneration, spine, low back pain, regeneration, clinical trials, minimally invasive surgery

## Abstract

Low back pain is critical health, social, and economic issue in modern societies. This disease is often associated with intervertebral disc degeneration; however, contemporary treatments are unable to target this underlying pathology to alleviate the pain symptoms. Cell therapy offers a promising novel therapeutic that, in theory, should be able to reduce low back pain through mitigating the degenerative disc environment. With the clinical development of cell therapeutics ongoing, this review aims to summarize reporting on the different clinical trials and assess the different regenerative strategies being undertaken to collectively obtain an impression on the potential safety and effectiveness of cell therapeutics against intervertebral disc-related diseases.

## 1. Introduction

**Figure 1:**
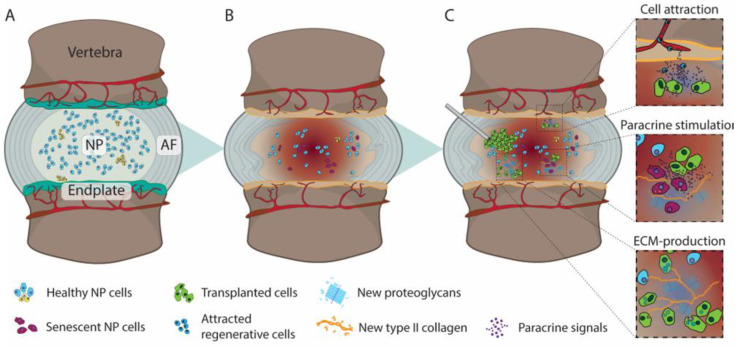
Illustration depicting (**A**) a healthy IVD with hydrated nucleus pulposus (NP) and organized annulus fibrosus (AF), (**B**) subsequent degenerative cascade resulting in AF disorganization loss of NP hydration, endplate vascularization, and disc height, and (**C**) Injection of de novo cells into the NP and their three proposed potential therapeutic mechanisms; i.e., (i) attraction of regenerative cells or limiting catabolic/inflammatory cells into the IVD, (ii) reactive and directing local cells to produce extracellular matrix (ECM), and (iii) integration into the IVD and contribution to ECM production directly.

Low back pain (LBP) and neck pain currently form the primary causes of global disability [1], and prevalence is likely to increase with a generally aging population, further imposing concerns on the socioeconomic affordability of healthcare and social security expenses [2,3]. Both disorders are generally associated with the intervertebral discs (IVD) being subdued to progressive age- and non-age-related degeneration [4]. IVD constitute the fibrocartilage tissues between each two vertebrae, capable of distributing complex loads along the spine. The IVD is composed of a highly hydrophilic core, the nucleus pulposus (NP), which is laterally enclosed by multiple collagenous lamellae, cumulatively termed the annulus fibrosus (AF) (Figure 1A). The IVD is connected to each vertebra with a thin hyaline cartilage layer, the endplate, which forms the primary source of nutrient, waste, and gas exchange for the predominantly avascular discs [5]. The IVD derives its function through a careful interplay of the high osmotic pressure engendered by the proteoglycan-rich NP, which is constricted by a stiff AF, jointly enabling the IVD to absorb relatively large forces while retaining flexibility. Tissue-specific cells are responsible for maintaining and remodeling the region-distinct extracellular matrix (ECM) to maintain the IVD and its biomechanical features. Although the etiology and progression of IVD degeneration remain somewhat obscure, it is generally associated with a progressive decline in cell numbers and a cellular switch toward a more catabolic and senescent state [6,7] (Figure 1B). Consequently, deteriorating the quality and organization of the ECM, thereby compromising the discs biomechanical limits [8]. Jointly, these changes engender an inflammatory environment, promoting immunogenic cell migration and potentially inducing blood vessel and neuronal ingrowth into the disc, thereby conceivably sensitizing the discs or inflaming regional spinal nerves [5,9]. Moreover, the progressive decline of the biomechanical sturdiness of the disc may allow AF tissue to bulge or burst, compressing neuronal and vascular tissues along the spine. Alternatively, the loss of water retention in the NP leads to a decline in disc height and mechanical features that further stress other spinal tissues, e.g., facet joints and tendons, to thereby be involved in the pathogenesis.

**Figure 2:**
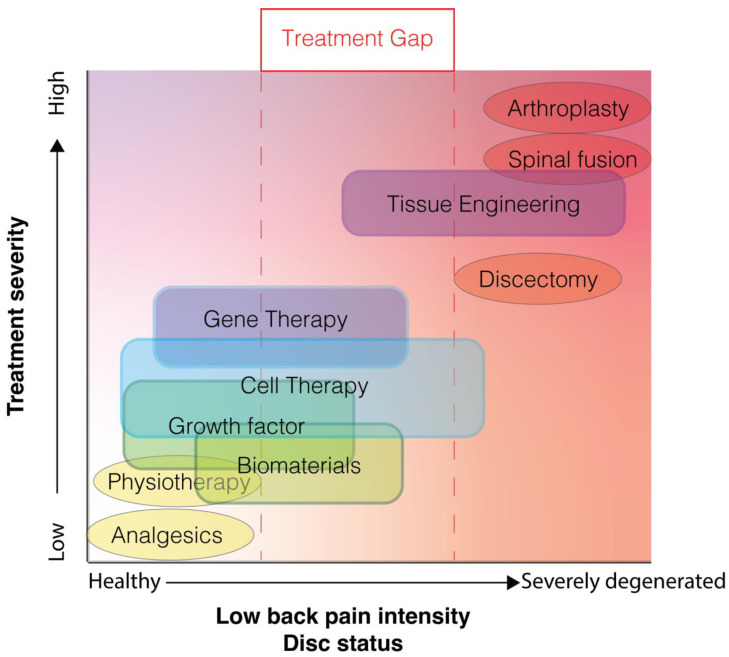
Illustrative plot depicting the contemporary treatment gap for low back pain associated with disc degeneration, in which only treatment options are available (oval) in either the mild or severe disc degeneration and low back pain range. New proposed techniques (blocks) likely will be most effective at different stages of degeneration and are likely less invasive than the surgical intervention currently employed.

Contemporary treatment strategies remain primarily palliative (Figure 2). Early-stage LBP is commonly treated with physiotherapy or the administration of analgesics [10]. Consequently, LBP has been indicated as the primary reason for non-cancer opioid prescription [11]. Nevertheless, high-quality evidence supporting the efficacy of these conservative therapies is severely lacking [10]. At later stages of LBP, surgical intervention may be employed. Generally, this involves either excision of protruding disc material in cases of disc herniation or complete removal of discs followed by arthroplasty or arthrodesis. Although these procedures are commonplace and rates are dramatically increasing [12,13], their efficacy remains largely controversial [14,15]. In addition, none of these interventions aim to resolve the degenerative cascade underlying the pathology, and furthermore, can trigger degenerative cascades in neighboring discs [16]. New strategies are being explored to limit, halt, and even reverse disc degeneration in an attempt to revitalize the disc’s composition and thus its biomechanical features, thereby resolving or preventing associated spinal disorders [17,18]. These include regenerative approaches, e.g., growth factor injection [19], gene therapy [20], tissue engineering [21,22], and biomaterial-applications [23], each being at different stages of development and presenting different levels of success in preclinical and/or clinical studies. Moreover, each strategy will likely be most effective at different stages of the degeneration cascade (Figure 2). One particular regenerative approach that has gained significant momentum in the recent decade is cell therapy [24,25]. Cell therapy involves the transplantation of additional cell populations into the IVD with the aim to either (i) directly impact IVD repair by repopulating the disc with de novo active cells to reestablish appropriate ECM production or (ii) indirectly induce IVD repair by stimulating or attracting regional cells to induce a more anabolic state, by for example tempering inflammation or to promote (re)initiation of IVD-ECM production by native cells (Figure 1C). The optimal strategy for restoring degenerative IVD in a clinic setting remains to be determined and is likely highly dependent on the degenerative state and disease indications. Multiple cell products and transplantation strategies have now been assessed in the clinical, showing promising results. Nevertheless, due to the accelerating speed of publication on this topic [24], new advancements in cellular therapy and new insights on potential limitations require continuous and careful review of progress in the field. Here we aim to assess the contemporary state and trends seen in the field of cell therapy in the clinic regarding safety and efficacy for cell-mediated therapy for the IVD. This review will discuss the main reported clinical trials, specifically focusing on the differences in cell products being examined. Moreover, we will supplement our review with our own experiences in cell-based therapeutics applied in a human condition.

## 2. Cell Therapy

Cell therapy is a therapeutic strategy in which generally living cells are introduced into the patient to replace or repair damaged tissue or otherwise alter endemic cell behavior. The most well-established form of cell therapy remains bone marrow transplantation for leukemia patients; however, since its first descriptions in 1968 [26,27], a wide range of other cell therapies have been postulated and examined, including regenerative strategies, cancer treatment, immunomodulation, or otherwise [28]. For the IVD, cellular therapeutics are commonly designed specifically for regenerative purposes, in which the transplanted or infused cells are expected to produce or stimulate the production of appropriate IVD-ECM or otherwise reduce the inflammatory and catabolic environment that typifies a degenerating IVD. As such, the cell product needs to either have the capacity to (1) survive and excel within the degenerating IVD to directly contribute to matrix product, (2) generate strong (paracrine)signaling able to promote an anabolic switch in native cells, or (3) support the recruitment of regenerative cells or otherwise limit the migration of fibrotic/catabolic cells into the IVD. Multiple in vivo animal studies have suggested the ability of transplanted cells to limit and sometimes even reverse the degeneration process [29,30,31]. Cell transplantation into the IVD is commonly employed through minimally invasive surgery, involving a percutaneous injection commonly through a 21- or 22-gauge needle [24] under fluoroscopic guidance into the IVD space. Cells upon transplantation can potentially be maintained in the IVD space due to the enclosed nature of the disc or, otherwise, might be retained by encapsulation in a hydrogel, tissue graft, or other carriers [32]. Multiple cell types have been examined as the agent engendering the repair, and these will be discussed in further detail below.

## 3. Mesenchymal Stromal Cells

Mesenchymal stromal cells (MSC), often falsely termed mesenchymal stem cells, usually involve a heterogeneous population of multipotent and more committed progenitor cells with relatively high proliferation capacity [33]. These cells can be sourced from multiple tissues; both are most often derived from bone marrow, adipose tissue, peripheral blood, or umbilical cords. Notably, however, the different MSC sources have been linked to differences in potency and differentiation inclinations [34,35,36]. MSC are of particular interest due to their easy accessibility and expandability [25]. Unlike other cell types, MSC can with relative ease be aspirated from healthy and young donors as well as from autologous sources [24]. Moreover, the MSC are characterized by their ability to differentiate toward a chondrogenic cell type, including the induction of a high rate of proteoglycan production [37,38,39]. Additionally, MSC possess an innate immunomodulatory capacity and could potentially limit the inflammatory environment of the IVD upon transplantation [40]. Nevertheless, their survival and capacity to strive in the IVD remain an aspect requiring careful examination [41,42]. For example, MSC subjected to IVD environmental factors have been shown to severely reduce proliferation and chondrogenic potency [43]. Although MSC have been shown capable of surviving and differentiating in the IVD of a range of animal models, their full NP cell phenotypical characteristics have not yet been reported [36]. Moreover, due to the unmatured nature of the MSC following transplantation and potential migration or leakage out of the IVD, they could potentially give rise to undesired differentiation and tissue formation, e.g., osteophyte as observed by Vadala et al. [44]. Finally, MSC have also been shown potent inducers of angiogenesis, particularly sourced from adipogenic tissue, which could further aggravate the degenerative cascade [45,46]. These practical benefits and opportunities should be carefully weighed out to the potential risks.

For human clinical trials, MSC are by far the most common cell type being examined. At current, MSC products are either sourced from adipose [47,48], bone marrow [49,50,51,52,53,54,55,56,57], or umbilical cord [58] tissues (Table 1) and applied either as an autologous or allogenic cell product. Two reports on adipose-derived MSC (AD-MSC) trials, involving a combined 18 patients, suggested that intradiscal injection at a 1-year follow-up was able to show a trend of [47] or significant [48] improvement in both visual analog pain scores (VAS) as well as Oswestry disability index (ODI). Interestingly, Piccirilli et al. [47] and Kumar et al. [48] reported an improvement in MRI signal intensity in 80% of examined discs and 30% of patients, respectively, suggesting to some extent the ability of the MSC to support IVD regeneration in some cases.

Bone marrow-derived MSC (BM-MSC) as an IVD therapeutic have been examined as an intradiscal injection product in 132 LBP patients [49,51,52,53,54,55,56,57] and as an intravenous infusion product for 31 ankylosing spondylitis patients [50]. In all studies that reported on pain or disability-related outcomes, at least a trend of improvement was observed (Table 1). For example, the non-controlled study of Orozco et al. [54] involving 10 patients resulted in significant VAS, ODI, and short-form 36 (SF-36) measurements. Of particular interest are the two controlled clinical trials. Noriega et al. [52,53] compared the transplantation of 25 × 10^6^ allogenic BM-MSC to a control involving a local paravertebral anesthesia injection. In their 1-year follow-up, the authors recorded a significant improvement in VAS and ODI values compared to baseline at higher rates than the control cohort. A more recent and larger randomized controlled clinical trial (RCT) study by Amirdelfan et al. [57] comparing high (18 × 10^6^) and low (6 × 10^6^) dosages of their allogenic “mesenchymal precursor cells” administration to a saline and hyaluronic acid vehicle control group. Their study showed a significantly enhanced improvement of LBP and ODI for cell-treated cohorts compared to control groups, with higher rates of responders. Though significant, the question can be raised regarding the clinical significance of these findings. Another consideration is underlined by the study of Noriega et al. [52,53], in which they reported a significant improvement in ODI and VAS values; however, according to the authors, this effect stemmed from a 40% portion of responders in their experimental cohort. MRI findings of the BM-MSC studies (Table 1) generally report at least maintenance of disc features, while most suggest a trend of improvement. Specifically, the work of Noriega et al. [52,53] highlighted a significant improvement of Pfirrmann grading for the cell-treated cohort, while their control group had a significant decline in Pfirrmann classification at 12 months compared to baseline. Orozco et al. similarly reported a significant improvement in MRI signal intensity in their treated IVD. On the contrary, the RCT of 60 patients by Amirdelfan et al. [57] failed to report a consistent improvement on MRI outcomes for their cell-treated cohorts. The intravenous infusion of allogenic BM-MSC, as reported by Wang et al. [50], was suggested to alleviate ankylosing spondylitis symptoms as observed through MRI.

Finally, the small case series by Pang et al. [58] applied allogenic umbilical cord-derived MSC (UC-MSC) in two patients. They reported a trend of VAS and ODI improvement 2 years follow-up with one of the two LBP patients presenting enhanced signal intensity on MRI compared to baseline.

Regarding safety outcomes of all MSC types, most of the studies reported no clear serious adverse events, only Amirdelfan et al. [57] reported 8 serious adverse events in their 60 cell-treated patients (1 involving discitis), compared with 4 of 40 patients in their control cohort. These events, however, did not include a severe immunogenic reaction. Of specific interest is the report of Garcia-Sancho et al. [52], complimenting in part the study of Noriega et al. [53], which assessed the influence of HLA matching regarding their allogenic MSC products. They found of the nine degenerative disc disease patients analyzed, none presented with HLA-targeted antibodies matching those of their MSC donors [52]. The lack of immunoreactivity, as suggested by the authors, might be found in the immunomodulatory potential of MSC or the immune privileged and enclosed nature of the IVD, though these suggestions remain highly speculative, especially considering the limited numbers analyzed. On the contrary, a small study by Henriksson et al. [49] applied iron sucrose-labeling to their transplanted BM-MSC for potential cell tracing. In their study, 4 of the 10 patients post MSC transplantation opted to undergo fusion surgery. As part of the fusion surgery, the IVD tissues were explanted, and iron sucrose-labeled cells were detected. Their assays revealed the presence of the transplanted MSC up to 28 months following transplantation. Moreover, additional staining suggested some but not all cells were apoptotic and were in close proximity to SOX9 and type II collagen-positive areas. Notably, however, was the detection of calcium deposits, suggesting early bone formation, in one of the four IVD explants. Again, raising some concern on the potential undesirable differentiation potential of MSC upon transplantation [44]. Though, these deposits were found in both iron sucrose-positive and -negative areas.

## 4. Nucleus Pulposus and Articular Cartilage-Derived Cells

NP cells form a heterogeneous cell population of native cells residing in the NP and can include highly differentiated rounded NP cells or less undifferentiated progenitor cells [6,73,74]. NP cells are the cells endemic to the IVD, and unlike the previously discussed MSC, they are specially adapted to survive and thrive within the harsh IVD environment [73,75]. Moreover, the chondrogenic NP cells are hallmarked by their high proteoglycan and type II collagen production rates [73]. Naturally, these cells possess the optimal cell type for the regeneration of the NP of the IVD and have been shown to retain in the IVD following transplantation in multiple animal studies [29,76]. Alternatively, other chondrogenic cells types have been suggested, specifically, chondrocytes from articular cartilage as well as hyaline cartilage tissues [43,77]. Similar to NP tissue, other articular cartilage sources are also avascular, and their cells are prone to high proteoglycan and type II collagen production and have thus been suggested and tested as an alternative cell source for IVD repair [36]. Notably, however, the rates of proteoglycan production have been shown to be much lower for articular chondrocytes compared to NP cells [78]. Although both cell types in preclinical studies are suggested to be very potent in supporting IVD repair, their applicability is mainly limited by practical consideration [24]. Specifically, NP and other cartilage sources have low accessibility, and often tissue sources that are obtainable are compromised by disease, age, or trauma [24]. Moreover, these chondrogenic cells types present a limited proliferation capacity and tend to lose their phenotypical features rapidly in vitro [6,79]. Although culture optimization strategies are being explored to enhance the expandability of these cell types [80,81,82].

Clinical studies applying either IVD-derived or articular cartilage-derived cells are less common and small in nature. In total, our review identified 5 separate studies (Table 1) involving 15 patients treated with articular chondrocytes and 220 treated with IVD-derived chondrocytes. A smaller case series by Mochida et al. [63] transplanted autologous NP cells, reactivated by MSC coculture ex vivo, as a strategy to limit degeneration progression in discs adjacent to fused IVD to prevent adjacent segment disease. We will discuss more on this trial in paragraph 6; nevertheless, overall, the procedure appeared safe and did not show any worsening of the adjacent segments on MRI observations. Similarly, work by Coric et al. [64], which employed juvenile articular chondrocytes, showed in a 1-year follow-up a significant improvement in pain rating as well as ODI and SF-36 outcomes for their 15 treated patients. Moreover, 10 out of 13 patients analyzed through MRI showed improvement on MRI. An RCT study by Meisel et al. [61,62] compared patients undergoing sequestrectomy to a cohort undergoing sequestrectomy followed by transplantation of autologous IVD-derived cells. Their study suggested a trend of improvement in VAS and ODI scores comparing the control to the experimental cohort. Moreover, MRI signal intensity was found significantly higher in the cell-treated cohort than the sequestrectomy-only group. An RCT by Tschugg et al. [59,60] involving autologous IVD-derived cell transplantation was compared to a control cohort only receiving hyaluronic acid-polyethylene glycol carrier. This phase I trial reported no clear evident worsening of disc MRI, but their report did not mention any clear enhancement for the cell-treated group compared to the carrier control group. Finally, a very recent RCT by Beall et al. [65,66] seeded “cells” in an NP allograft and compared the treatment effects to a placebo control and conservative treatment cohort. Conservative treatment recipients were allowed to crossover at 3 months post transplantation to the allograft cohort if outcomes were unsatisfactory. Their 1-year follow-up study revealed a significant reduction in pain and ODI values for the allograft and crossover group; however, these changes appear similar to the reduction seen in their placebo cohort, resulting in a lack of statistical significance. Nevertheless, a post-hoc stratification analysis by Hunter et al. [67] highlighted that when only patients were considered below the age of 42 years, a statistically significant improvement was observed for mean change in ODI comparing allograft and crossover cohorts separately to the placebo control. Moreover, responder rates for both active groups were significantly higher than the placebo for both ODI (≥10 points) and VAS (≥50%) outcomes. Thereby highlighting the likely need for careful patient selection [83].

Regarding safety outcomes, none of the studies reported any serious adverse events, with the exception of the Vivex Biomedical RCT [65,66], which involved 11 serious adverse events in their allograft cohort and 1 in their crossover group. Six of these were considered potentially related to the treatment and included bacteremia and osteomyelitis.

## 5. Combination Strategies and Other Cell Types

The final cell group being discussed here involves the transplantation of cells not clearly definable as either MSC or chondrogenic cells. For example, the first reported human clinical trial of Haufe et al. [72] applied hematopoietic stem cells (HSC) as their transplantation product. In the 10 LBP patients treated with HSC, none reported improvement in pain outcomes in their 1-year follow-up.

Alternatively, Comella et al. [68] applied the transplantation of stromal vascular fraction (SVF) combined with platelet-rich plasma, a.k.a. PRP. Stromal vascular fraction involves adipose tissue that is processed to remove the majority of adipose cells, connective tissue, and blood, leaving behind a vastly heterogeneous population of cells, including AD-MSC as well as endothelial cells, immunogenic cells, smooth muscle cells, and pericytes [84]. The SVF with PRP transplantation suggested a trend of enhanced VAS pain ratings, but only a minimal improvement in disability outcomes for their 15 patients after 1 year. No serious adverse events were reported.

Another cell product being reported examined by Pettine et al. [69,70] was autologous bone marrow concentrate (BMC) injections. BMC involves the centrifugation of bone marrow aspirates, resulting in a mixture containing a wide range of cell types, including hematopoietic cells, adipocytes, stromal cells, platelets, macrophages, fibroblasts, osteoblasts, osteoclasts, endothelial cells, lymphocytes, MSC, as well as chondrogenic progenitor cells, just to mention a few [85]. In addition to cellular constituents, it is also rich in numerous bioactive factors, e.g., TGF-β, FGF, IGF-1, etc., that are suspected of supporting regeneration through their anabolic and anti-inflammatory effects [85]. The study by Pettine et al. [69,70] suggested that their transplantation product could result in a significant improvement of VAS and ODI scores, as well as a Pfirrmann-grade improvement for 40% of their participants. Interestingly, they reported a relationship between the rate of colony-forming units derived from their BMC products with improvement. No serious adverse events were reported. Finally, a case report by Subach et al. [71] reports on a single patient that presented at their clinic, following treatment from an outside provider, which included transplantation of bone marrow aspirate, blood plasma, and adipose tissue-derived transplantation products. The patient was diagnosed with cauda equina syndrome, involving discitis with osteomyelitis by methicillin-resistant *Staphylococcus epidermidis*, which was finally resolved following decompression surgery, arthrodesis, and antibiotic treatment [71].

With the above, it appears that the general effects of the less well-defined cell products are limited; only the study of Pettine et al. [69,70] was able to present a clear significant improvement for their participant cohort. As these BMC and SVF products are composed of a complex combination of different cells as well as growth factors and potentially ECM components, it will be challenging to determine which specific components are responsible for any clinical outcome observed. Moreover, batch-to-batch differences might form a hurdle regarding quality control [25,86]. Alternatively, the combination of growth factors with the different cell types has been suggested to enable a synergistic effect; however, whether such effects hold true in a human IVD remains to be determined [85].

## 6. Our Experience

**Figure 3:**
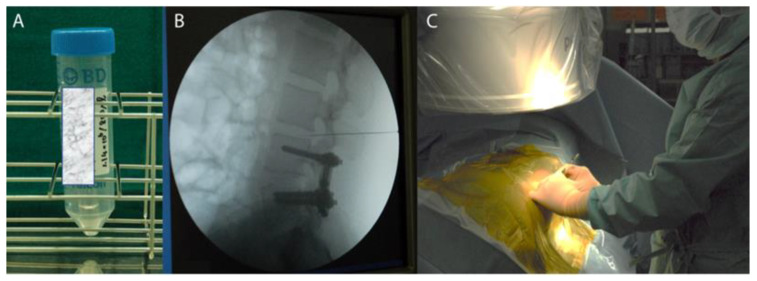
(**A**) NP cell suspension after coculture with autologous MSC for 1 week. (**B**) Cells are injected into adjacent segments with moderate degeneration next to the fused disc via fluoroscopic visualization. (**C**) A needle injectable access provides an advantage compared to other target organs for the application of regenerative medicine as the patient does not require surgical exposure for receiving their regenerative medicinal product.

After preclinical studies, a clinical study was conducted to examine the possibility of preventing the development of adjacent disc disease in patients who underwent spinal fusion [63]. Cells were isolated from degenerated vertebral bodies excised for fusion and then reactivated in vitro by co-culturing with autologous MSC. Mochida et al. [63] reported a three-year observational study in 9 patients aged 20–29 years who had Pfirrmann-grade III disc degeneration at the level adjacent to the level scheduled for posterior lumbar intervertebral fusion. Viable NP cells derived from the tissues excised from the fused disc were co-cultured in direct contact with autologous BM-MSC. One million activated NP cells were transplanted into the degenerated disc adjacent to the fused level 7 days after their first fusion surgery (Figure 3). No adverse effects were observed during the 3-year follow-up period. MRI did not show any detrimental effects to the transplanted discs and revealed a mild improvement in one case. No cases reported any LBP post cell transplantation confirming the safety of activated NP cell transplantation, and the findings suggested the minimal efficacy of this treatment to slow the further degeneration of human IVD [63].

In 2019, a clinical trial of cell transplantation for LBP patients with underlying lumbar disc degeneration had finally started in Japan. A phase I/II, Multicenter, 2-dose (low-dose, high-dose) double-blinded trial to evaluate the safety and efficacy of cell therapy products developed by DiscGenics Inc. (Salt Lake City, UT, USA) is currently under investigation (NCT03955315). DiscGenics^TM^ cell products are composed of allogeneic NP progenitor cells isolated from fresh cadaveric disc tissues. Unlike conventional cell therapy products that use autologous cells as therapeutic materials, therapies using allogeneic cells have the potential to be industrially mass produced, reducing treatment costs and quality variations and increasing market introduction [25].

## 7. Discussion

Notably, a wide range of cellular and cell-derived products for intradiscal transplantation is being assessed to treat intervertebral disc degeneration. All of them are still in the initial phases of clinical development. Each of the different products was able to alleviate pain or improve disability symptoms to some extent; however, the overall quality of evidence is limited. This is in part due to the small cohorts’ sizes, lack of appropriate control groups, as well as limited follow-up periods and outcome measures. Moreover, noticeably, the reporting on these clinical trials is also of rather poor quality. Critical comparisons and statistical analysis are lacking from multiple of the trial reports, and critical details on cell product or transplantation methods are lacking or remain ambiguous; for example, as presented in Table 1, multiple studies do not clearly define the cell concentrations or volumes used for their transplantation product. We would strongly encourage researchers, peer-reviewers, and editors, to be more stringent toward authors and their published work to ensure these critical details are made publicly available to allow for a better understanding of the cell transplantation products and the resulting outcomes of the studies. With these caveats in mind, we do believe that the collection of data supports the notion that an intradiscal injection of cells for the treatment of LBP associated with a degenerative IVD is generally safe, as no serious adverse events were reported in any of the studies. Only one case report [71] and one RCT participant [57] were diagnosed with discitis following cell transplantation. It remains difficult to determine whether the infection was introduced as part of the procedure, cell product, or otherwise. However, surgical intervention always comes with a risk of infection; however, this risk should not be disregarded as cautioned by Subach et al. [71].

With regard to efficacy, the current data do not provide a clear encompassing conclusion in this regard. As the trials all include different cell products, outcome measures, and patient cohorts, direct comparison remains difficult. There is some suggestion that cell transplantation can enhance MRI hydration values and improve Pfirrmann classifications, as was observed in a portion of patients following MSC transplantation cohorts in the work of Piccirilli [47], Kumar [48], Yoshikawa [51], Noriega [53], and Pang [58], as well as Coric [64], Meisel [61,62], and Mochida [63] for chondrogenic cells, and Pettine [69,70] using their BMC product. In particular, the work of Noriega et al. [53] is of interest as their study showed significant Pfirrmann improvement for the cell-treated group, while their control cohort, on the other hand, presented significant worsening. On the other hand, however, the largest study of Amirdelfan et al. [57] was unable to report clear MRI improvements, and of the studies that did show improvement, these generally involved only a portion of responding participants. The topic of cells being able to regenerate the IVD matrix is a rather controversial item in the field [87]. As, concern has been raised regarding the limited nutrient and oxygen availability in the IVD, in particular degenerating IVD [31,87,88]. Moreover, the number of cells transplanted has been shown to affect clinical outcomes in preclinical studies [29,89]. Interestingly, the work of Elabd et al. [56] and Pettine et al. [69,70] suggested their higher concentration of cells or colony-forming units, respectively, enhanced outcomes. On the other hand, Kumar et al. [48] and Amirdelfan et al. [57] did not observe clear differences in outcomes between higher and lower cell dosages.

Although MRI observations are interesting, as they present some quantitative data regarding potential tissue regeneration, these do not form the primary outcome measure of interest. The prime aim of cellular therapeutics is to alleviate LBP in the participant and mitigate disability. In this regard, all reports involving MSC or chondrogenic cells reported at least a trend of improvement in pain outcomes and most a trend for disability improvements (Table 1). However, the majority of studies did not include a control cohort, and as can be seen in the mesoblast study [57], a vehicle or placebo injection can already trigger some improvement in pain and disability outcomes. From the four studies that included a control group, only the work of Noriega et al. [53] and Amirdelfan et al. [57] were able to report a significantly greater improvement for the cell-treated cohort compared to their controls. Nevertheless, though statistical significance was mentioned, it remains unclear whether these differences are clinically significant, long-lasting, and economically impactful. Combining all data, it is evident that the clinical translation of cellular therapeutics is still in the early stages of development. Included trials mainly involved prospective pilot studies or phase I/II trials, with only one study being a phase II trial. Larger scale, placebo-controlled, randomized, multicenter, and efficacy-focused studies will prove critical to really grasp the potential impact of cell therapeutics in alleviating LBP and potentially regenerative degenerating IVD. In addition, a small selection of larger RCT is ongoing or has been completed. For example, the DiscGenics^TM^ trial in the USA (NCT03347708) and Japan (NCT03955315) assessed NP-derived cells, as well as the BioRestorative Therapies RCT (NCT04042844) applying bone marrow-derived mononuclear cells combined with platelet lysate. As well as the ongoing phase III mesoblast trial (NCT02412735) and the terminated phase II NuQu^®^ (NCT01771471) trial. The results of these studies are highly anticipated. In addition, a careful, cost-effectiveness analysis will prove critical in order to confirm the financial feasibility of cellular therapeutics [25].

Finally, some noticeable cell types have not been reported as of yet. Firstly, although IVD-derived cells have been examined, currently, no studies have examined the potential of IVD-specific progenitor or stem cells [74,90]. Resupplying the IVD with innate stem or progenitor cells could form an infinite source of young and active NP cells, which thus could engender long-term cell sources for IVD repair. The initial discovery of Tie2/GD2-expressing NP progenitor cells has been suggested to be critical for IVD maintenance and has since been shown to be effective in a mouse model for IVD tissue maintenance [6,74,91]. In addition, induced pluripotent stem cells (iPSC) have shown potent cell sources to create an infinite amount of potent NP cells and could form a promising source for future cell products [92,93]. Finally, all regenerative strategies are primarily focused on restoring the NP. Cell products designed for AF or endplate restoration might provide an alternative treatment strategy or be applicable to specific patient indications [30,36,94,95]. In addition, the applicability of cell products will likely be best applied for specific patient populations and disease indications. For example, the report of Hunter et al. highlighted that their cell therapeutic was primarily effective for younger (<42 years old) individuals [67]. Similarly, the trial of Pettine et al. also found that patients above 40 years showed remarkably different outcomes based on colony-forming units, while younger patients did not [69,70]. Other indications, such as obesity, diabetes, general physical activity, etc., could also be potential confounding factors that potentially influence the therapeutic outcomes of cell products. In addition, specific IVD features and their optimal strategy can be considered; for example, whether the need exists for a fully enclosed IVD or uncompromized endplates, which could be confirmed via MRI modalities. Hopefully, future large-scale clinical trials will be able to provide some indications to which patient populations are most likely to benefit from cellular therapeutics against LBP [24,25].

## 8. Conclusions

A slight reduction in the number of back pain patients, associated medical care, or back pain intensity can make a significant impact on socioeconomic costs, and benefits can be achieved for many patients. Degenerative disc disease is deeply involved in the onset and progression of spinal diseases such as lumbar pain, herniated disk, spinal canal stenosis, and spondylolisthesis. Yet, a radical cure has not yet been established. Under these circumstances, cell therapy is considered a promising means for coping with degeneration of IVD, and various clinical trials are being conducted all over the world. With new advances in the regulatory framework for marketing approval of medical drugs and careful consideration of the marketability of these cell therapies, these products will likely be available for health care providers and patients in the near future.

## Figures and Tables

**Table 1:** Overview of reported clinical trials, case series, and case reports on cell transplantation for IVD repair.

	Trial Design							Outcomes			
	Sponsor, Study [Ref]	Trial Type	Control	Product (Type)	Dose (Cell/mL)	Cohort (n)	FU (y)	Pain	Disability	MRI	SAE
Mesenchymal Stromal Cells	Piccirilli [47]	Case series	None	AD-MSC (Autologous)	ns/~1 mL	8	1	Trend of VAS improvement	Trend of ODI improvement	80% of disc regained signal intensity	None
	Kumar [48]	PhaseI/IIa trial	None	AD-MSC (Autologous)	20 × 10^6^/ 2 mL HA	5	1	Significantly enhanced VAS	Significantly enhanced ODI and SF-36	3/10 patients presented enhanced intensity	None
					40 × 10^6^/ 2 mL HA	5					
	Henriksson [49]	Prospective study	None	BM-MSC (Autologous)	1 × 10^6^/ns	10	<3	-	-	-	Calcium deposits observed in 1/4 patients
	Wang [50]	Prospective study	None	BM-MSC (Allogenic)	4 × 1 × 10^6^ (/kg BW)/10 mL *	31	<1	-	-	Ankylosing spondylitis features mitigated	None
	Elabd [56]	Unspecified	None	BM-MSC (Autologous)	31 (±14) × 10^6^/0.25–1 mL PL	5	6	-	Trend of improvement in strength and mobility	-	None
	Yoshikawa [51]	Case series	None	BM-MSC (Autologous)	ns/ns	2	2	Trend of VAS improvement	Trend of amended JOA scores	Trend increased signal intensity	None
	Citospin/TerCel, Noriega [52,53]	RCT, blinded, phase I/II	Paravertebral muscle anesthesia	BM-MSC (Allogenic)	25 × 10^6^/ 2 mL	12	1	Significant VAS improvement, significantly higher than control	Significant ODI improvement, significantly higher than control	Significantly enhanced Pfirrmann grading while worsening in control	None
	ITRT, Orozco [54]	Phase I/II trial	None	BM-MSC (Autologous)	10 (±5) × 10^6^/ns	10	1	Significant VAS improvement	Significant ODI and SF-36 improvements	Significant increase in signal intensity	None
	Regenexx, Centeno [55,56]	Prospective study	None	BM-MSC + PL (Autologous)	1–3 × 10^6^/10–20% PL 1–2 mL, +3–5 mL PL (epidural)	33	7	Significant NPS improvement	Trend of FRI score improvement	85% showed reduction in disc bulge size	None
	Mesoblast, Amirdelfan [57]	RCT, blinded, phase II	(1) Saline injection (2) HA injection	BM-MSC (Allogenic)	6 × 10^6^/2 mL HA	30	2	Significant VAS improvement, significantly higher than sham control	Significant ODI improvement, significantly higher than sham control	No clear difference in Pfirrmann grades	8/60 SAE compared to 4/40 in control, 1 case of discitis
					18 × 10^6^/2 mL HA	30					
	Pang [58]	Case series	None	UC-MSC (Allogenic)	10 × 10^6^/1 mL	2	2	Trend of VAS improvement	Trend of ODI improvement	1/2 patients showed increase in signal intensity	None
Chondrogenic cells	NOVOCART^®^, Tschugg [59,60]	RCT, blinded phase I/II	PEG-HA injection	IVD cells (Autologous)	ns/0.5–2 mL PEG-HA	12	<1	-	-	No improvements reported	None
	Meisel [61,62]	RCT	Sequestrectomy only	IVD cells + Sequestrectomy (Autologous)	ns/ns	22	>5	Trend of VAS improvement compared to control	Trend of ODI improvement compared to control	Significant improvement signal intensity compared to control	None
	Mochida [63]	Case series	None	IVD cells (Autologous)	1 × 10^6^/0.7 mL	9	3	Trend of LBP subscale improvement	Trend of JOA improvement	Signal intensity maintained. 1/9 showed Pfirrmann-grade improvement	None
	NuQu^®^, Coric [64]	Phase I trial	None	AC (Allogenic)	10–20 × 10^6^/1–2 mL Fibrin	15	1	Significant NRS improvement	Significant ODI and SF-36 improvements	10/13 patients presented MRI ameliorations	None
	Vivex Biomedical, Beall [65,66,67]	RCT, crossover study	(1) placebo, (2) conservative care	“Spine-derived” cells in NP tissue allograft (Allogenic)	>6 × 10^6^/1.25–1.75 mL NP allograft	140 (+37) **	1	Significant VAS improvement, not different from placebo group	Significant ODI improvement, not different from placebo group, unless stratified for younger patients (<42 y)	-	11 SAE in allograft and 1 in crossover cohort, 6 considered treatment related; including osteomyelitis and bacteremia
Other/Combined	Bioheart, Comella [68]	Prospective study	None	SVF + PRP (Autologous)	30–60 × 10^6^/1–3 mL PRP	15	1	Trend of VAS and pain rating improvements	Minimal improvements in disability and QoL scores	-	None
	Pettine [69,70]	Prospective study	None	BMC (Autologous)	1–2 × 242–363 × 10^6^/2–3 mL	26	3	Significant VAS improvements	Significant ODI improvements	40% present Pfirrmann-grade improvement	None
	Subach [71]	Case report	None	BMA + Adipose tissue + Plasma (Autologous)	ns/3 mL	1	1	-	-	-	Disc extrusion, discitis with osteomyelitis requiring in emergency surgery
	Haufe [72]	Prospective study	None	HSC (Autologous)	ns/ns	10	1	No pain improvement	-	-	None

* Cells administered per intravenous infusion as opposed to an intradiscal injection, ** following crossover. Abbreviations: AC; articular cartilage cells, AD; adipose derived, BM; bone marrow derived, BMA; bone marrow aspirate, BMC; bone marrow concentrate, BW; body weight, FRI; functional index rating, FU; maximum follow-up time, HA; hyaluronic acid, HSC; hematopoietic stem cells, ITRT; Instituto de Terapia Regenerativa Tissular, IVD; intervertebral disc, JOA; Japanese orthopaedic association, MRI; magnetic resonance imaging, NP; nucleus pulposus, NPS; numerical pain score, ns; not specified, ODI; Oswestry disability index, PEG; polyethylene glycol, PL; platelet lysate, PRP; platelet-rich plasma, QoL; quality of life, RCT: randomized controlled clinical trial SAE; serious adverse events, SF; short form, SVF; stromal vascular fraction, UC; umbilical cord derived, VAS; visual analog (pain) score.

## Data Availability

Not applicable.
